# Soil invertebrates of coniferous forests along a gradient of air pollution (Komi Republic)

**DOI:** 10.3897/BDJ.9.e75586

**Published:** 2021-11-17

**Authors:** Alla Kolesnikova, Tatyana Konakova, Anastasia Taskaeva, Alexey Kudrin

**Affiliations:** 1 Institut of Biology Komi SC UrD RAS, Syktyvkar, Russia Institut of Biology Komi SC UrD RAS Syktyvkar Russia

**Keywords:** sampling event, Lumbricidae, Julidae, Polyzoniidae, Lithobiidae, Carabidae, Staphylinidae, Elateridae, Curculionidae, Collembola, Nematoda, pine and spruce forests, pulp and paper industry, Komi Republic

## Abstract

**Background:**

The role of soil invertebrates in the cycle of substances, soil-forming processes and the provision of ecosystem services is undeniable. Therefore, soil invertebrates are valuable in bioindication studies. Comprehensive research of soil invertebrates in the production area of Mondi Syktyvkar JSC as the largest pulp and paper enterprise in the European part of Russia was initiated in 2003. A huge amount of data about composition, abundance and structure of soil macro- and mesofauna along an impact gradient was accumulated during the period from 2003 to 2019 years. These data can be used to study local biodiversity, monitor the state of soil invertebrate communities and assess the impact of the pulp and paper industry on the environment.

**New information:**

Datasets here presented include information from a monitoring programme for soil invertebrates that inhabit coniferous forests in the production area of Mondi Syktyvkar JSC (Komi Republic). The assemblages' structure of macrofauna, collembolans and nematodes are described. Information on the number of individuals of springtail species, nematodes genera and macrofauna taxa is given. A total of 11146 sampling events of macrofauna, 6673 sampling events of Collembola, and 2592 sampling events of Nematoda are recorded along a gradient of air pollution from pulp and paper industry emissions.

## Introduction

Previously, we have already published data on the occurrence of soil invertebrates on the European North-East of Russia (Konakova et al. 2020a, Konakova et al. 2020b). It is shown that soil invertebrates are characterised by high abundance and diversity in the study area. The structure of invertebrate communities reflects changes in soil-ecological factors. The response of invertebrates to ecosystems changes under anthropogenic impact has importance for monitoring of the state of soils (Lavelle et al. 2006, Zvereva and Kozlov 2010). Intensive industrial development in Europe has a significant effect on natural ecosystems. Although the main production centres of the pulp and paper industry are located in the countries of North America and Northern Europe, there areonly poor data about such anthropogenic effects on the soil invertebrate diversity in literature (Dolgin et al. 2012). It is shown that air pollution by emissions from chemical enterprises causes changes in the abundance, biomass, diversity, structure and spatial distribution of soil fauna (Rusek and Marshall 2000, Kuznetsova and Ivanova 2020, Nesterkov et al. 2020). The technologies used in the pulp and paper industry at the end of the 20^th^ century have significantly changed today. New and more advanced technological processes have appeared (Jaworska et al. 2020). Works aimed at reducing of pollutants emissions into the environment have been carried out at the Mondi Syktyvkar JSC since 2002 (Baturina et al. 2020). An improvement in the vital state of forest stands was noted. There is no pronounced negative impact of the enterprise on the soils (Robakidze and Torlopova 2018). The ongoing reduction of emissions from the modernised enterprises makes it possible to analyse the natural recovery of soil fauna (Vorobeichik et al. 2019). There are practically no long-term observations of the soil invertebrates response to environmental changes under industrial impact. Dataset about nematodes is the initial study to understanding diversity and abundance of this group in the production area of pulp and paper industry enterprise. Datasets about large soil invertebrates and collembolans allow us to assess the changes that have occurred in the reduction of industrial emissions.

## General description

### Purpose

The main goal was to describe datasets on sampling events of macrofauna (Konakova and Kolesnikova 2021), Collembola (Taskaeva 2021) and Nematoda (Kudrin 2021) in coniferous forests along a gradient of air pollution (Komi Republic). The objectives of this study are to include data of observations for the last 18 years and to provide a basis for monitoring research of soil invertebrate communities in impacted areas of aero-technogenic emissions from the pulp and paper industry.

## Project description

### Title

Soil invertebrates of coniferous forests along a gradient of air pollution in Komi Republic - "Russia 2021"

### Personnel

Alla Kolesnikova, Tatyana Konakova, Anastasia Taskaeva, Alexey Kudrin

### Study area description

**Figure 1.**
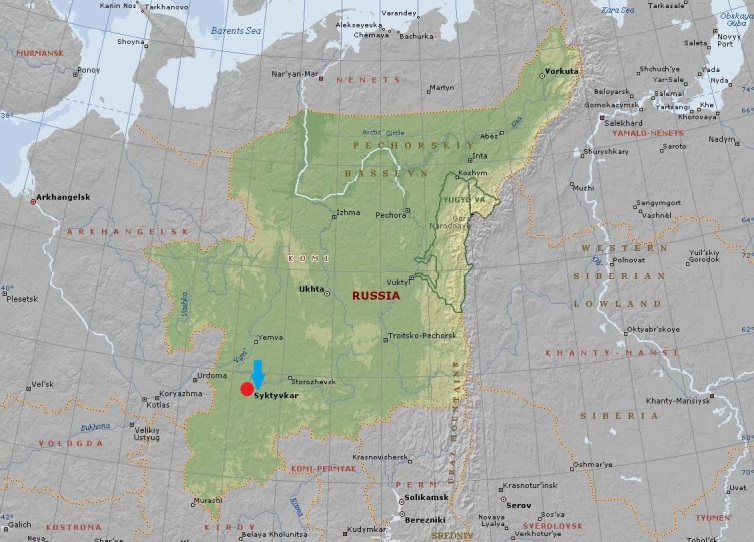
Study area (European part of Russia, Komi Republic, Syktyvkar).

**Figure 2.**
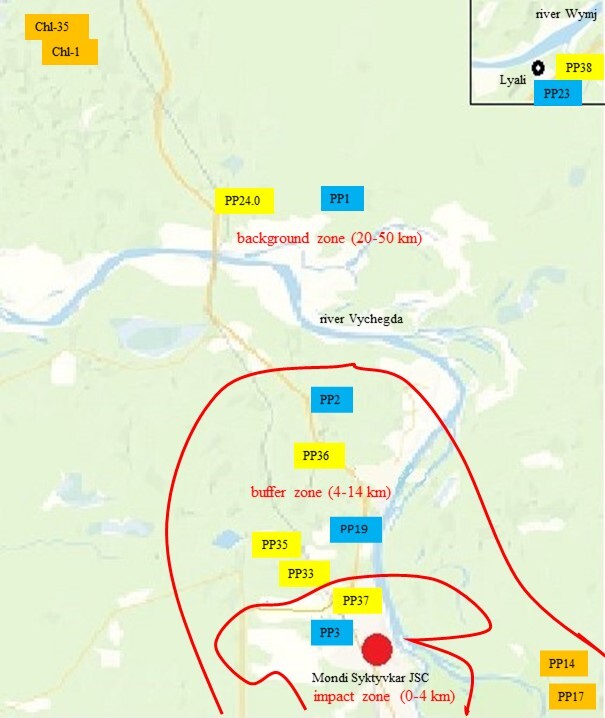
The map-scheme of study area (lichen pine forest is marked orange, bilberry pine forest - blue, bilbery spruce forest - yellow colour).

The study was carried out in the European part of Russia, Komi Republic, vicinity of Syktyvkar City (Fig. 1). Soil macro- and mesofauna at the production area of Mondi Syktyvkar JSC was reviewed. Mondi Syktyvkar JSC is the largest pulp and paper industry in the European part of Russia and also a source of industrial emissions of various pollutants into the air basin. It has been operating since 1964. The emissions include sulphur and nitrogen oxides, hydrogen sulphide, sulphur anhydride, mineral dust containing calcium and sodium carbonates and sulphides (Torlopova and Robakidze 2012). Since 2002, the enterprise has been carrying out large-scale work aimed at reducing the impact of airborne man-made emissions on the environment. According to the company's public environmental reports, the total amount of emissions in 1999 was 31, in 2006 - 20 and in 2015 - 10 thousand tonnes (Robakidze and Torlopova 2018). In the area of the enterprise emissions, three zones with different levels of technogenic load were conditionally identified (Table 1, Fig. 2) according to the calculations of the pollution index (Zc) i.e. the sum of the excess concentrations of substances in the melt water of snow samples from the contaminated area relative to the concentrations of substances in background samples. There are no clear boundaries in space between different emissions' zones and there are some transitional strips of a greater or lesser extent where the values of index Zc can be higher or lower for the denoted emission area. The impact zone (zone of the strong impact of emissions) includes the territory of the working zone of the pulp and paper enterprise within a radius of 3.5 km, where the levels of technogenic load exceed the background onesby 100-150 times and values of pollution index (Zc) are 129 – 391. The territory, with (Zc) values over 128, is classified as an extremely dangerous pollution area. The buffer zone consists of two sub-zones - areas with significant and moderate impacts of emissions. The first subzone is located concentrically at a distance of up to 6 km from the emission centre along the line of the prevailing wind direction. Here, the levels of technogenic load exceed the background onesby 20-100 times and values of pollution index (Zc) are 65 – 128. In the second subzone (at a distance of up to 14 km), the levels of technogenic load exceed the background ones by 4-20 times and values of pollution index (Zc) are 33 – 64. Unification of territories with significant and moderate impact of emissions into one buffer zone is possible, since the reduction of emissions from the enterprise contributes to the shift in the boundaries of these conventionally- allocated zones. The territory, with (Zc) values over 32, is classified as a dangerous pollution area. The values of (Zc) are 0 – 32 at a distance from the boundaries of the buffer zone to the boundaries of the background zone. So, the absence of any emissions impact is allowed. The analysis of emissions dispersion from Mondi Syktyvkar JSC showed that the distribution of the main emissions components occurs at a distance of up to 18 km. Therefore, coniferous forests located at a distance of 22–50 km from the enterprise were chosen as sites of the background zone (Baturina et al. 2020).

### Funding

This project was supported by The Ministry of Science and Education of Russian Federation АААА-А17-117112850235-2 "Distribution, systematics and spatial organisation of fauna and animals population in taiga and tundra landscapes and ecosystems at the Northeast European Russia"; agreement with JSC "Mondi SLPK" № 45-2018/180405 "Assessment of long-term impact of Mondi Syktyvkar JSC on the biological diversity in the production area".

## Sampling methods

### Study extent

We built our database by compiling sampling events of soil invertebrates, based on an exhaustive search in published and unpublished authors' sources, as well as from the Zoological Museum of the Institute of Biology Komi SC UB RAS. The data paper is based on three datasets. The datasets about macrofauna and collembolans provides temporal data on the number of their individuals in soil cores collected in pine and spruce forests of the production area of the pulp and paper industry enterprise. The dataset about macrofauna also contains the material collected by the pitfall traps and information on the age stages (larvae and imago) of invertebrates. Nematodes were collected in pine and spruce forests of the production area of Mondi Syktyvkar JSC only in 2018-2019, so these data are not temporal. The dataset about Nematoda provides information on the number of their individuals in soil cores. Three datasets have differences in data presentation. We have combined them into one data paper, since a comprehensive study of the effect on soil invertebrates from the pulp and paper industry has not previously been carried out.

### Sampling description

The standard methods of soil zoology were applied. Sampling and pitfall trapping were carried out in pine and spruce forests of the impact, buffer and background zones of the enterprise production area.

Two sampling methods were used for collection of macrofauna at each site.

1. Soil sampling method. About 8-10 soil cores were randomly taken to a depth of 5-10 cm at each site. A quadrangular frame of 25 × 25 cm or 10 × 10 cm was used for sampling. Actively moving animals were placed into containers with 70% alcohol as a fixative. Further, in laboratory conditions, the extraction of invertebrates from soil cores into 70% alcohol was carried out in the funnels.

2. Pitfall trapping. Pitfall traps of the same type (plastic glasses, depth 10 cm, diameter 12.0 cm, salt water (30% vol.) and formalin (5% vol.) as a fixative) were used at each site. The traps were emptied once per 7-10 days; all collected invertebrates were preserved in 95% alcohol for subsequent identification with a binocular microscope.

One sampling method was used for Collembola collection. In each site, 10 soil samples were taken. Samples were taken to the full depth with division into two layers - litter and mineral horizons; in further analysis, the data on the layers were combined. Extraction was carried out according to the generally- accepted method using Berlese – Tulgren funnels in 96% alcohol for 7–10 days - the time sufficient to achieve an air-dry soil state. Collembolans from the samples were sorted under a binocular microscope. All individuals were mounted in slides in Phoera liquid according to a standard procedure (Potapov and Kuznetsova 2011).

The soil sampling method also used for Nematoda collection. Soil samples were collected from the organic and mineral soil horizons. In pine forests, eight soil cores (5 × 5 cm, 10 cm depth) were taken at each site. In spruce forests, five soil cores (5 × 5 cm, 10 cm depth) were taken at each site. Nematodes were extracted from 50 g of fresh soil using the modified Baermann Method (Ruess 1995). Extraction lasted for 48 h. Extracted nematodes were euthanised at 60°C and then preserved in 4% formaldehyde.

The material includes data collected during the period of modernisation and reduction of emissions of the enterprise. Thus, the real changes in soil invertebrate communities can be estimated.

### Quality control

The data were collected by specialists from the Institute of Biology of the Komi Scientific Center of the Ural Branch of the Russian Academy of Sciences. Lumbricidae, Lithobiidae, Julidae, Polyzoniidae, Coleoptera (Carabidae, Staphylinidae, Elateridae, Curculionidae, Ostomatidae) and some Hemiptera were identified to species by specialists from the Institute of Biology. All specimens of Collembola and Nematoda were also identified to species and genera, respectively, by specialists from the Institute of Biology. Different taxonomic keys were used for identification of large soil invertebrates (Lohse 1964, Gurjeva and Kryzhanovskij 1965, Lohse 1974, Vsevolodova-Perel 1997, Andersson et al. 2005), Collembola (Fjellberg 1998, Potapov 2001, Fjellberg 2007) and Nematoda (Jairajpuri and Ahmad 1992, Bongers 1994, Brzeski 1998). The material (invertebrates fixed in alcohol and formalin, on preparations and in entomological collections) is kept in the Zoological Museum of the Institute of Biology. On each slide and/or label, the following fields were filled out: “Collection date”, “Locality” (with geographic coordinates), “Habitat”, “Collector name” and “Determined by” (identification).

## Geographic coverage

### Description

The study area is located in the middle taiga zone of Komi Republic. The Mondi Syktyvkar JSC production area is located on the Vychegda River bank in the vicinity of Syktyvkar City. There are arrays of spruce and pine forests in the research area.

**Coordinates**: 61°40′35″ Latitude 1 to 62°27′58″ Latitude 2; 50°48′35″ Longitude 1 to 50°66′76″ Longitude 2.

## Taxonomic coverage

### Description

**Figure 3.**
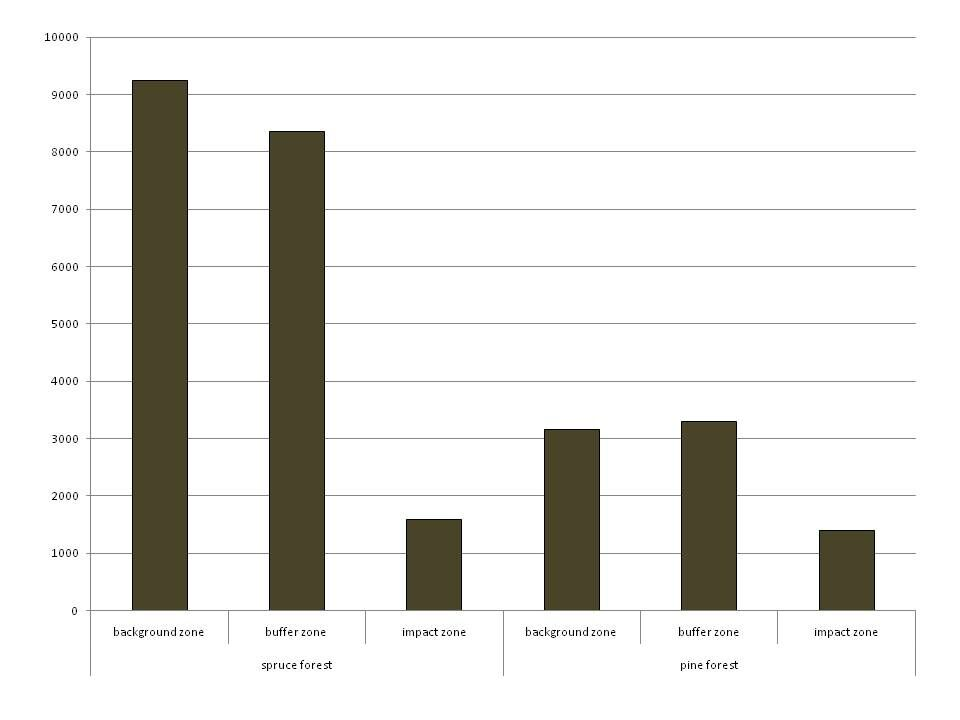
Nematodes abundance (ind./100 g, along the ordinate axis) along the pollution gradient.

**Figure 4.**
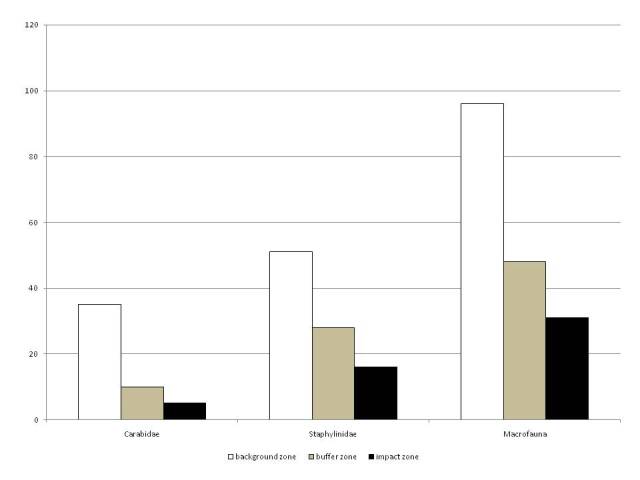
The number of macrofauna species in spruce forests along the pollution gradient.

**Figure 5.**
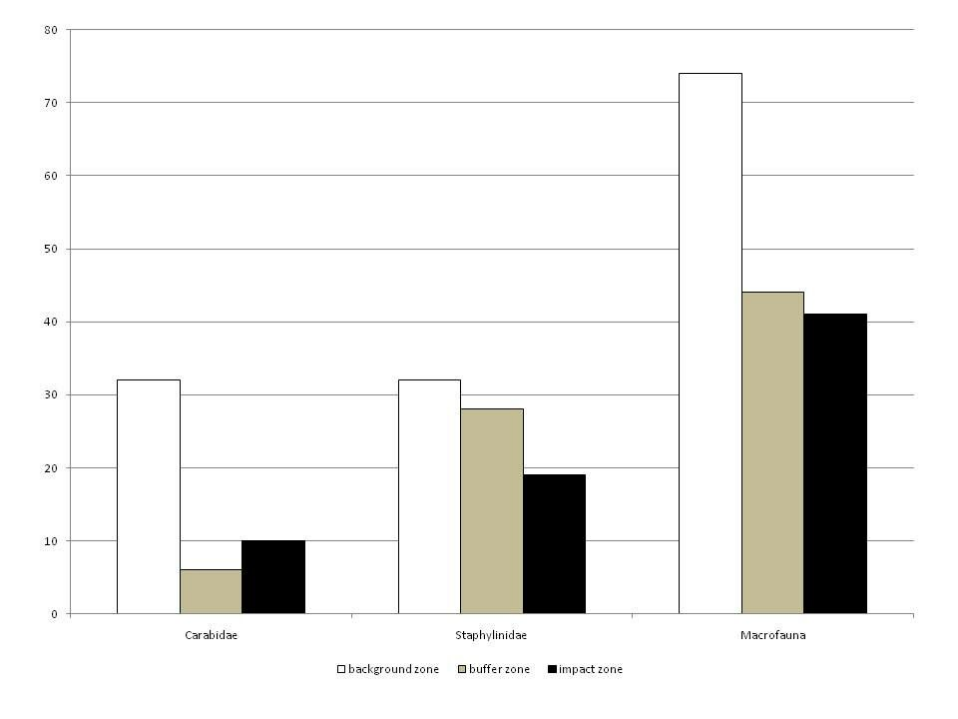
The number of macrofauna species in pine forests along the pollution gradient.

The datasets contain all of the information obtained during the sampling for macrofauna, Collembola and Nematoda. A decrease of nematodes abundance (Fig. 3) and the decline in the species richness of macrofauna (Figs 4, 5) was noted in spruce and pine forests along the pollution gradient. There are changes in the abundance and number of Collembola species along the pollution gradient before and after emission reduction (Table 2).

### Taxa included

**Table:** 

Rank	Scientific Name	Common Name
phylum	Nematoda	nematodes
phylum	Annelida	
class	Clitellata	
order	Crassiclitellata	
family	Lumbricidae	earthworms
phylum	Arthropoda	arthropods
class	Diplopoda	
family	Julidae	
family	Polyzoniidae	
class	Chilopoda	centipedes
family	Lithobiidae	centipedes
class	Insecta	insects
order	Collembola	springtails
order	Coleoptera	beetles
family	Carabidae	ground beetles
family	Staphylinidae	rove beetles
family	Elateridae	click beetles
family	Curculionidae	weevils

## Temporal coverage

### Notes

At the present time, the following periods are covered for large soil invertebrates: 2007-2010, 2018 years (bilberry pine forests), June, July 2010 year (lichen-pine forests), 2003-2004, 2006, 2008-2010, 2019 years (bilberry spruce forests). The following periods are covered for Collembola: 2007-2009, 2018 years (bilberry pine forests), June, July 2010 year (lichen pine forests), June, July 2003 and 2019 years (bilberry spruce forests). The following periods are covered for Nematoda: 2018 year (bilberry pine forests), 2019 year (bilberry spruce forests).

## Collection data

### Collection name

Soil invertebrates_Mondi Syktyvkar JSC

### Specimen preservation method

alcohol, formalin, dried

## Usage licence

### Usage licence

Creative Commons Public Domain Waiver (CC-Zero)

### IP rights notes

This work is licensed under a Creative Commons Attribution (CC-BY) 4.0 License.

## Data resources

### Data package title

Soil invertebrates of coniferous forests along a gradient of air pollution (Komi Republic).

### Resource link

https://www.gbif.org/dataset/d52fad79-9ba7-432c-a595-1cede017e70d https://www.gbif.org/dataset/3dd06b2d-fbe5-4000-9001-5efe3da4aa5f https://www.gbif.org/dataset/0f17aeea-fe0b-40e4-9e85-0095bbeb5f1f

### Alternative identifiers

d52fad79-9ba7-432c-a595-1cede017e70d, http://ib.komisc.ru:8088/ipt/resource?r=sfm; 3dd06b2d-fbe5-4000-9001-5efe3da4aa5f, http://ib.komisc.ru:8088/ipt/resource?r=lpk; 0f17aeea-fe0b-40e4-9e85-0095bbeb5f1f, http://ib.komisc.ru:8088/ipt/resource?r=nematodes_lpk

### Number of data sets

3

### Data set 1.

#### Data set name

Large soil invertebrates of coniferous forests along a gradient of air pollution: temporal series of the data (Komi Republic).

#### Data format

Darwin Core Archive format

#### Number of columns

43

#### Character set

UTF-8

#### Download URL

d52fad79-9ba7-432c-a595-1cede017e70d, http://ib.komisc.ru:8088/ipt/resource?r=sfm

#### Description

The dataset includes two tables related by the eventID field – Events and Associated occurrences.

**Data set 1.** 

Column label	Column description
eventID (Event Core)	An identifier for the event (layer) http://rs.tdwg.org/dwc/terms/eventID
eventDate (Event Core)	Field data collection date (dd/mm/yyyy) http://rs.tdwg.org/dwc/terms/eventDate
habitat (Event Core)	A category or description of the habitat http://rs.tdwg.org/dwc/terms/habitat
samplingProtocol (Event Core)	Sampling protocol http://rs.tdwg.org/dwc/terms/samplingProtocol
samplingEffort (Event Core)	The amount of effort expended during an Event http://rs.tdwg.org/dwc/terms/samplingEffort
sampleSizeValue (Event Core)	Size of the sampling core http://rs.tdwg.org/dwc/terms/sampleSizeValue
sampleSizeUnit (Event Core)	The unit of measurement of the size sampling core http://rs.tdwg.org/dwc/terms/sampleSizeUnit
locationID (Event Core)	An identifier for the place of field data collection http://rs.tdwg.org/dwc/terms/locationID
Continent (Event Core)	The name of the continent in which the Location occurs http://rs.tdwg.org/dwc/terms/continent
country (Event Core)	Country name (Russian Federation) http://rs.tdwg.org/dwc/terms/country
countryCode (Event Core)	The standard code for the Russian Federation according ISO 3166-1-alpha-2 (RU) http://rs.tdwg.org/dwc/terms/countryCode
stateProvince (Event Core)	Region (‘oblast’) name. The first-level administrative division http://rs.tdwg.org/dwc/terms/stateProvince
Locality (Event Core)	The specific description of the place http://rs.tdwg.org/dwc/terms/locality
decimalLatitude (Event Core)	The geographic latitude in decimal degrees of the geographic centre of the data sampling place http://rs.tdwg.org/dwc/terms/decimalLatitude
decimalLongitude (Event Core)	The geographic longitude in decimal degrees of the geographic centre of the data sampling place http://rs.tdwg.org/dwc/terms/decimalLongitude
geodeticDatum (Event Core)	Spatial reference system (SRS) upon which the geographic coordinates given in decimalLatitude and decimalLongitude are based http://rs.tdwg.org/dwc/terms/geodeticDatum
georeferencedBy (Event Core)	A list (concatenated and separated) of names of people, groups or organisations who determined the georeference (spatial representation) for the Location http://rs.tdwg.org/dwc/terms/georeferencedBy
eventID (Occurrence Extension)	An identifier for the event (layer) http://rs.tdwg.org/dwc/terms/eventID
occurrenceID (Occurrence Extension)	An identifier for the record http://rs.tdwg.org/dwc/terms/occurrenceID
basisOfRecord (Occurrence Extension)	Basis of the record (PreservedSpecimen) http://rs.tdwg.org/dwc/terms/basisOfRecord
recordedBy (Occurrence Extension)	List of persons who collected field data http://rs.tdwg.org/dwc/terms/recordedBy
individualCount (Occurrence Extension)	The number of individuals represented in the event http://rs.tdwg.org/dwc/terms/individualCount
occurrenceStatus (Occurrence Extension)	A statement about the presence/absence of a Taxon at a Location http://rs.tdwg.org/dwc/terms/occurrenceStatus
lifeStage (Occurrence Extension)	The life stage of individuals. Here it is used for juvenile individuals indicated http://rs.tdwg.org/dwc/terms/lifeStage
organismQuantityType (Occurrence Extension)	The type of quantification system used for the quantity of organisms http://rs.tdwg.org/dwc/terms/organismQuantityType
associatedReferences (Occurrence Extension)	A list (concatenated and separated) of identifiers (publication, bibliographic reference, global unique identifier, URI) of literature associated with the Occurrence http://rs.tdwg.org/dwc/terms/associatedReferences
kingdom (Occurrence Extension)	The full scientific name of the kingdom http://rs.tdwg.org/dwc/terms/kingdom
phylum (Occurrence Extension)	The full scientific name of the phylum http://rs.tdwg.org/dwc/terms/phylum
Class (Occurrence Extension)	The full scientific name of the class http://rs.tdwg.org/dwc/terms/class
order (Occurrence Extension)	The full scientific name of the order http://rs.tdwg.org/dwc/terms/order
family (Occurrence Extension)	The full scientific name of the family http://rs.tdwg.org/dwc/terms/family
Genus (Occurrence Extension)	The full scientific name of the genus http://rs.tdwg.org/dwc/terms/genus
specificEpithet (Occurrence Extension)	The name of the first or species epithet of the scientificName http://rs.tdwg.org/dwc/terms/specificEpithet
infraspecificEpithet (Occurrence Extension)	The name of the lowest or terminal infraspecific epithet of the scientificName, excluding any rank designation http://rs.tdwg.org/dwc/terms/infraspecificEpithet
scientificNameAuthorship (Occurrence Extension)	The authorship information for the scientificName formatted according to the conventions of the applicable nomenclaturalCode http://rs.tdwg.org/dwc/terms/scientificNameAuthorship
namePublishedInYear (Occurrence Extension)	The four-digit year in which the scientificName was published http://rs.tdwg.org/dwc/terms/namePublishedInYear
scientificName (Occurrence Extension)	The full scientific name, with authorship and date information if known http://rs.tdwg.org/dwc/terms/scientificName
taxonRank (Occurrence Extension)	The taxonomic rank http://rs.tdwg.org/dwc/terms/taxonRank
identifiedBy (Occurrence Extension)	List of persons who identified collected invertebrates http://rs.tdwg.org/dwc/iri/identifiedBy
year (Event Core)	The year in which the Event occurred, according to the Common Era Calendar http://rs.tdwg.org/dwc/terms/year
month (Event Core)	The month in which the Event occurred http://rs.tdwg.org/dwc/terms/month
day (Event Core)	The day of the month on which the Event occurred http://rs.tdwg.org/dwc/terms/day
coordinateUncertaintyInMetres	The horizontal distance (in metres) from the given decimalLatitude and decimalLongitude describing the smallest circle containing the whole of the Location https://dwc.tdwg.org/terms/#dwc:coordinateUncertaintyInMeters

### Data set 2.

#### Data set name

Collembola of coniferous forests along a gradient of air pollution: temporal series of the data (Komi Republic)

#### Data format

Darwin Core Archive format

#### Number of columns

41

#### Character set

UTF-8

#### Download URL

3dd06b2d-fbe5-4000-9001-5efe3da4aa5f, http://ib.komisc.ru:8088/ipt/resource?r=lpk

#### Description

The dataset includes two tables related by the eventID field – Events and Associated occurrences.

**Data set 2.** 

Column label	Column description
eventID (Event Core)	An identifier for the event (layer) http://rs.tdwg.org/dwc/terms/eventID
eventDate (Event Core)	Field data collection date (dd/mm/yyyy) http://rs.tdwg.org/dwc/terms/eventDate
habitat (Event Core)	A category or description of the habitat http://rs.tdwg.org/dwc/terms/habitat
samplingProtocol (Event Core)	Sampling protocol http://rs.tdwg.org/dwc/terms/samplingProtocol
samplingEffort (Event Core)	The amount of effort expended during an Event http://rs.tdwg.org/dwc/terms/samplingEffort
sampleSizeValue (Event Core)	Size of the sampling core http://rs.tdwg.org/dwc/terms/sampleSizeValue
sampleSizeUnit (Event Core)	The unit of measurement of the size sampling core http://rs.tdwg.org/dwc/terms/sampleSizeUnit
locationID (Event Core)	An identifier for the place of field data collection http://rs.tdwg.org/dwc/terms/locationID
Continent (Event Core)	The name of the continent in which the Location occurs http://rs.tdwg.org/dwc/terms/continent
country (Event Core)	Country name (Russian Federation) http://rs.tdwg.org/dwc/terms/country
countryCode (Event Core)	The standard code for the Russian Federation according ISO 3166-1-alpha-2 (RU) http://rs.tdwg.org/dwc/terms/countryCode
stateProvince (Event Core)	Region (‘oblast’) name. The first-level administrative division http://rs.tdwg.org/dwc/terms/stateProvince
Locality (Event Core)	The specific description of the place http://rs.tdwg.org/dwc/terms/locality
decimalLatitude (Event Core)	The geographic latitude in decimal degrees of the geographic centre of the data sampling place http://rs.tdwg.org/dwc/terms/decimalLatitude
decimalLongitude (Event Core)	The geographic longitude in decimal degrees of the geographic centre of the data sampling place http://rs.tdwg.org/dwc/terms/decimalLongitude
geodeticDatum (Event Core)	Spatial reference system (SRS) upon which the geographic coordinates given in decimalLatitude and decimalLongitude are based http://rs.tdwg.org/dwc/terms/geodeticDatum
georeferencedBy (Event Core)	A list (concatenated and separated) of names of people, groups or organiations who determined the georeference (spatial representation) for the Locationhttp://rs.tdwg.org/dwc/terms/georeferencedBy
eventID (Occurrence Extension)	An identifier for the event (layer) http://rs.tdwg.org/dwc/terms/eventID
occurrenceID (Occurrence Extension)	An identifier for the record http://rs.tdwg.org/dwc/terms/occurrenceID
basisOfRecord (Occurrence Extension)	Basis of the record (PreservedSpecimen) http://rs.tdwg.org/dwc/terms/basisOfRecord
recordedBy (Occurrence Extension)	List of persons who collected field data http://rs.tdwg.org/dwc/terms/recordedBy
individualCount (Occurrence Extension)	The number of individuals represented in the event http://rs.tdwg.org/dwc/terms/individualCount
occurrenceStatus (Occurrence Extension)	A statement about the presence/absence of a Taxon at a Location http://rs.tdwg.org/dwc/terms/occurrenceStatus
organismQuantityType (Occurrence Extension)	The type of quantification system used for the quantity of organisms http://rs.tdwg.org/dwc/terms/organismQuantityType
associatedReferences (Occurrence Extension)	A list (concatenated and separated) of identifiers (publication, bibliographic reference, global unique identifier, URI) of literature associated with the Occurrence http://rs.tdwg.org/dwc/terms/associatedReferences
kingdom (Occurrence Extension)	The full scientific name of the kingdom http://rs.tdwg.org/dwc/terms/kingdom
Phylum (Occurrence Extension)	The full scientific name of the phylum http://rs.tdwg.org/dwc/terms/phylum
Class (Occurrence Extension)	The full scientific name of the class http://rs.tdwg.org/dwc/terms/class
order (Occurrence Extension)	The full scientific name of the order http://rs.tdwg.org/dwc/terms/order
Family (Occurrence Extension)	The full scientific name of the family http://rs.tdwg.org/dwc/terms/family
Genus (Occurrence Extension)	The full scientific name of the genus http://rs.tdwg.org/dwc/terms/genus
specificEpithet (Occurrence Extension)	The name of the first or species epithet of the scientificName http://rs.tdwg.org/dwc/terms/specificEpithet
scientificNameAuthorship (Occurrence Extension)	The authorship information for the scientificName formatted according to the conventions of the applicable nomenclaturalCode http://rs.tdwg.org/dwc/terms/scientificNameAuthorship
namePublishedInYear (Occurrence Extension)	The four-digit year in which the scientificName was published http://rs.tdwg.org/dwc/terms/namePublishedInYear
scientificName (Occurrence Extension)	The full scientific name, with authorship and date information, if known http://rs.tdwg.org/dwc/terms/scientificName
taxonRank (Occurrence Extension)	The taxonomic rank http://rs.tdwg.org/dwc/terms/taxonRank
identifiedBy (Occurrence Extension)	List of persons who identified collected Collembola http://rs.tdwg.org/dwc/iri/identifiedBy
year (Event Core)	The year in which the Event occurred, according to the Common Era Calendar http://rs.tdwg.org/dwc/terms/year
month (Event Core)	The month in which the Event occurred http://rs.tdwg.org/dwc/terms/month
day (Event Core)	The day of the month on which the Event occurred http://rs.tdwg.org/dwc/terms/day
coordinateUncertaintyInMetres	The horizontal distance (in metres) from the given decimalLatitude and decimalLongitude describing the smallest circle containing the whole of the Location https://dwc.tdwg.org/terms/#dwc:coordinateUncertaintyInMeters

### Data set 3.

#### Data set name

Soil nematodes of coniferous forests along a gradient of air pollution (Komi Republic).

#### Data format

Darwin Core Archive format

#### Number of columns

41

#### Character set

UTF-8

#### Download URL

0f17aeea-fe0b-40e4-9e85-0095bbeb5f1f, http://ib.komisc.ru:8088/ipt/resource?r=nematodes_lpk

#### Description

The dataset includes two tables related by the eventID field – Events and Associated occurrences.

**Data set 3.** 

Column label	Column description
eventID (Event Core)	An identifier for the event (layer) https://dwc.tdwg.org/terms/#dwc:eventID
eventDate (Event Core)	Field data collection date (dd/mm/yyyy) http://rs.tdwg.org/dwc/terms/eventDate
habitat (Event Core)	A category or description of the habitat http://rs.tdwg.org/dwc/terms/habitat
samplingProtocol (Event Core)	Sampling protocol http://rs.tdwg.org/dwc/terms/samplingProtocol
samplingEffort (Event Core)	The amount of effort expended during an Event http://rs.tdwg.org/dwc/terms/samplingEffort
sampleSizeValue (Event Core)	Size of the sampling core http://rs.tdwg.org/dwc/terms/sampleSizeValue
sampleSizeUnit (Event Core)	The unit of measurement of the size sampling core http://rs.tdwg.org/dwc/terms/sampleSizeUnit
locationID (Event Core)	An identifier for the place of field data collection http://rs.tdwg.org/dwc/terms/locationID
Continent (Event Core)	The name of the continent in which the Location occurs http://rs.tdwg.org/dwc/terms/continent
country (Event Core)	Country name (Russian Federation) http://rs.tdwg.org/dwc/terms/country
countryCode (Event Core)	The standard code for the Russian Federation according ISO 3166-1-alpha-2 (RU) http://rs.tdwg.org/dwc/terms/countryCode
stateProvince (Event Core)	Region (‘oblast’) name. The first-level administrative division http://rs.tdwg.org/dwc/terms/stateProvince
Locality (Event Core)	The specific description of the place http://rs.tdwg.org/dwc/terms/locality
decimalLatitude (Event Core)	The geographic latitude in decimal degrees of the geographic centre of the data sampling place http://rs.tdwg.org/dwc/terms/decimalLatitude
decimalLongitude (Event Core)	The geographic longitude in decimal degrees of the geographic centre of the data sampling place http://rs.tdwg.org/dwc/terms/decimalLongitude
geodeticDatum (Event Core)	Spatial reference system (SRS) upon which the geographic coordinates given in decimalLatitude and decimalLongitude are based http://rs.tdwg.org/dwc/terms/geodeticDatum
georeferencedBy (Event Core)	A list (concatenated and separated) of names of people, groups or organisations who determined the georeference (spatial representation) for the Locationhttp://rs.tdwg.org/dwc/terms/georeferencedBy
eventID (Occurrence Extension)	An identifier for the event (layer) http://rs.tdwg.org/dwc/terms/eventID
occurrenceID (Occurrence Extension)	An identifier for the record http://rs.tdwg.org/dwc/terms/occurrenceID
basisOfRecord (Occurrence Extension)	Basis of the record (PreservedSpecimen) http://rs.tdwg.org/dwc/terms/basisOfRecord
recordedBy (Occurrence Extension)	List of persons who collected field data http://rs.tdwg.org/dwc/terms/recordedBy
individualCount (Occurrence Extension)	The number of individuals represented in the event http://rs.tdwg.org/dwc/terms/individualCount
occurrenceStatus (Occurrence Extension)	A statement about the presence/absence of a Taxon at a Location http://rs.tdwg.org/dwc/terms/occurrenceStatus
organismQuantityType (Occurrence Extension)	The type of quantification system used for the quantity of organisms http://rs.tdwg.org/dwc/terms/organismQuantityType
associatedReferences (Occurrence Extension)	A list (concatenated and separated) of identifiers (publication, bibliographic reference, global unique identifier, URI) of literature associated with the Occurrence http://rs.tdwg.org/dwc/terms/associatedReferences
kingdom (Occurrence Extension)	The full scientific name of the kingdom http://rs.tdwg.org/dwc/terms/kingdom
Phylum (Occurrence Extension)	The full scientific name of the phylum http://rs.tdwg.org/dwc/terms/phylum
Class (Occurrence Extension)	The full scientific name of the class http://rs.tdwg.org/dwc/terms/class
order (Occurrence Extension)	The full scientific name of the order http://rs.tdwg.org/dwc/terms/order
Family (Occurrence Extension)	The full scientific name of the family http://rs.tdwg.org/dwc/terms/family
Genus (Occurrence Extension)	The full scientific name of the genus http://rs.tdwg.org/dwc/terms/genus
scientificNameAuthorship (Occurrence Extension)	The authorship information for the scientificName formatted according to the conventions of the applicable nomenclaturalCode http://rs.tdwg.org/dwc/terms/scientificNameAuthorship
namePublishedInYear (Occurrence Extension)	The four-digit year in which the scientificName was published http://rs.tdwg.org/dwc/terms/namePublishedInYear
scientificName (Occurrence Extension)	The full scientific name, with authorship and date information, if known http://rs.tdwg.org/dwc/terms/scientificName
taxonRank (Occurrence Extension)	The taxonomic rank http://rs.tdwg.org/dwc/terms/taxonRank
identifiedBy (Occurrence Extension)	List of persons who identified collected Nematoda http://rs.tdwg.org/dwc/iri/identifiedBy
associatedReferences (Occurrence Extension)	A list (concatenated and separated) of identifiers (publication, bibliographic reference, global unique identifier, URI) of literature associated with the Occurrence http://rs.tdwg.org/dwc/terms/associatedReferences
year (Event Core)	The year in which the Event occurred, according to the Common Era Calendar http://rs.tdwg.org/dwc/terms/year
month (Event Core)	The month in which the Event occurred http://rs.tdwg.org/dwc/terms/month
day (Event Core)	The day of the month on which the Event occurred http://rs.tdwg.org/dwc/terms/day
coordinateUncertaintyInMetres	The horizontal distance (in metres) from the given decimalLatitude and decimalLongitude describing the smallest circle containing the whole of the Location https://dwc.tdwg.org/terms/#dwc:coordinateUncertaintyInMeters

## Figures and Tables

**Table 1.** Monitoring plots (PP), with distance from the emission centre of Mondi Syktyvkar JSC.

Type of forest	Zone of technogenic load		
	impact (0-4 km)	buffer (4-14 km)	background (20-50 km)
Lichen pine forest		PP14 (11.0)	ChL-1 (38.0)
		PP17 (11.2)	ChL-35 (38.5)
Bilberry pine forest	PP3 (1.3)	PP19 (6.5)	PP1 (22.0)
		PP2 (12.7)	PP23 (48.5)
Bilberry spruce forest	PP37 (3.5)	PP33 (4.3)	PP24.0 (24.0)
		PP35 (5.3)	PP38 (50.0)
		PP36 (10.0)	

**Table 2.** Abundance and species number of Collembola along the pollution gradient.

Parameters	Pollution zone, year					
	Background		Buffer		Impact	
	2007-2009^p^ 2003^s^	2018^p^ 2019^s^	2007-2009^p^ 2003^s^	2018^p^ 2019^s^	2007-2009^p^ 2003^s^	2018^p^ 2019^s^
Pine (p) forests						
Abundance (thousand ind./m^2^)	17.9 ± 5.2	10.2 ± 2.3	58.4 ± 14.8	15.6 ± 2.5	25.8 ± 3.3	7.4 ± 1.2
Number of species	14	26	33	34	26	33
Spruce (s) forests						
Abundance (thousand ind./m^2^)	75.0 ± 6.5	25.7 ± 4.4	38.0 ± 3.6	19.5 ± 4.0	47.8 ± 6.4	16.8 ± 2.9
Number of species	41	29	30	29	23	24
